# Kato-Katz versus urine POC-CCA for the diagnosis of *Schistosoma mansoni* in preschool-aged children in Homa Bay County, Kenya

**DOI:** 10.1007/s00436-025-08467-3

**Published:** 2025-02-21

**Authors:** Phyllis Munyiva Isaiah, Bryan Nyawanda, Collins Okoyo, Peter Steinmann

**Affiliations:** 1 Swiss Tropical and Public Health Institute, Allschwil, Switzerland; 2 University of Basel, Basel, Switzerland; 3 Centre for Global Health Research, Kenya Medical Research Institute, Kisumu, Kenya; 4 Eastern and Southern Africa Centre of International Parasite Control, Kenya Medical Research Institute, Nairobi, Kenya; 5 Department of Epidemiology, Statistics and Informatics, Kenya Medical Research Institute, Nairobi, Kenya

**Keywords:** Schistosomiasis, Preschool-aged children, POC-CCA, Kato-Katz

## Abstract

The diagnosis of schistosomiasis in preschool-aged children (PSAC) is challenging. The point-of-care circulating cathodic antigen (POC-CCA) assay offers potential improvements in diagnostic access in hard-to-reach areas over the conventional Kato-Katz (KK) test. This study compared the diagnostic accuracy of KK versus POC-CCA in stool and urine samples from PSAC in a PSAC population residing in a *S. mansoni* endemic hard-to-reach area in Kenya. Using combined positive results of both KK and POC-CCA tests as the “gold standard,” the overall sensitivity of KK (57.6%; 95% CI, 49.1–65.8) was significantly lower than that of POC-CCA (73.2%; 95% CI, 65.0–80.4); ${\chi }_{m}^{2}$= 5.88, *p* = 0.015. There was moderate agreement between KK and the combined results (*k* = 0.599, *p* < 0.001, concordance 80.9%) and a substantial agreement between POC-CCA and the combined results (*k* = 0.753, *p* < 0.001, concordance 88.1%). POC-CCA is a more sensitive diagnostic tool for schistosomiasis surveillance in PSAC compared to KK, particularly in hard-to-reach endemic areas.

## Introduction

Schistosomiasis remains a highly prevalent parasitic infection, causing significant economic and public health impacts, particularly in African impoverished communities (Cisse et al. 2021). *Schistosoma mansoni*, the causative agent for intestinal schistosomiasis, is responsible for one-third of Africa’s 192 million caseload (Cisse et al. 2021). Preschool-aged children (PSAC) are traditionally considered a low-risk group for *S. mansoni*, partly due to missing data. Therefore, they have been so far largely excluded from control programs, perpetuating health inequality (Cisse et al. 2021). Hence, accurate and reliable diagnosis is crucial for effective surveillance and disease control planning.

Currently, there is no gold standard test for diagnosing intestinal schistosomiasis. The World Health Organization (WHO) recommends the Kato-Katz (KK) method for intestinal schistosomiasis control programs (World Health Organization 2022). The test material is relatively cheap, and it is used in field surveys to diagnose schistosomiasis, make treatment decisions, and monitor program effectiveness (Stothard et al. 2014). Despite the test’s high specificity, its sensitivity in a single stool sample examination is limited by day-to-day variation in egg excretion rates and the non-homogeneous distribution of eggs in the stool (Stothard et al. 2014). This is particularly accentuated in populations with high proportions of light-intensity infections, resulting in a significant underestimation of prevalence and over-estimation of treatment efficacy (Straily et al. 2022). Further, trained laboratory staff, functioning microscopes, and usually also electricity are required to perform KK tests (Straily et al. 2022). In addition, stool collection for KK testing is burdensome, particularly when targeting PSAC living in remote areas. Moreover, as diarrheal diseases are prevalent in PSAC, diarrheic stool samples represent a critical challenge in preparing stool smears for the KK test (Straily et al. 2022). These challenges are exacerbated in remote and hard-to-reach locations.

The WHO recognizes KK’s limitations and calls for improvement and validation of point-of-care circulating cathodic antigen (POC-CCA) assay for *S. mansoni* in its 2030 strategic plan for ending schistosomiasis as a public health problem (World Health Organization 2022). This rapid test detects the presence of worm antigens in the urine of the host and can differentiate active from past infections (Yin et al. 2021). Unlike sensitive KK testing, POC-CCA testing requires only one urine sample. Urine collection among PSAC is generally more culturally acceptable, less invasive, and significantly quicker compared to stool samples (Straily et al. 2022) making it a more feasible option in hard-to-reach remote areas with limited healthcare infrastructure. In addition, there is less variation in the daily antigen excretion levels in urine, and the reading of POC-CCA results is straightforward (Mazigo et al. 2018). The tests can, in principle, also be used to estimate the intensity of infection, determine therapeutic responses, and ascertain the persistence of infection (Mazigo et al. 2018). Previous studies have shown high sensitivity and specificity in the detection of *S. mansoni* when using POC-CCA compared to KK (Fuss et al. 2018).

Based on these advantages, several studies suggest that POC-CCA is a promising test for the diagnosis of *S. mansoni*, including in PSAC and in remote areas (Yin et al. 2021). Our study compared the diagnostic accuracy of KK versus POC-CCA in fecal and urine samples of PSAC from hard-to-reach *S*. *mansoni*-endemic areas of Homa Bay County in Western Kenya.

## Materials and methods

This cross-sectional study was conducted between February and March 2024 and covered 6 islands of Lake Victoria, located in Suba North and Suba West Sub-Counties, Homabay County, Kenya. It included 319 PSAC aged 2–5 years.

Following informed consent by parents/caregivers, single stool and urine samples were collected in clean, separate containers from each enrolled PSAC. Samples were transported to Mbita Sub-County Hospital and examined for the presence of *S. mansoni* eggs using the KK technique (two smears per sample) (Katz et al. 1972) and parasite circulating antigen by POC-CCA (batch: 230,515,042), as per the manufacturer’s instructions (Rapid Medical Diagnostics 2019). Specifically, tests were scored positive or negative after 20 min, and invalid tests were repeated.

The performance of KK and POC-CCA was evaluated using combined positive results of both tests as the “gold standard,” following an evaluation approach used previously which assumed an (almost) 100% specificity for POC-CCA (Coulibaly et al. 2013). Statistical difference in sensitivity and specificity was calculated using McNemar’s chi-square test. Concordance between the diagnostic techniques was determined using the kappa statistics and interpreted as per Landis and Koch’s classification (Landis and Koch 1977).

## Results and discussion

In our study population, a total of 319 individuals provided both stool and urine samples. The overall prevalence of *S. mansoni* was 26.0% (95% CI, 21.3–31.2) by KK and 32.5% (95% CI, 25.7–41.1) by POC-CCA. Of the 83 infected PSAC by KK, 59.0% (95% CI, 47.7–69.7) had a light infection, 32.5% (95% CI, 22.7–43.7) a moderate infection, and 8.4% (95% CI, 3.5–16.6) a heavy infection.

True positive results were obtained from 83 (26.0%) and 101 (32.4%) individuals for KK and POC-CCA testing respectively, while true negative results were obtained from 175 (54.9%) and 174 (55.8%) individuals for KK and POC-CCA, respectively. Further, 61 discrepancies (0 false positives and 61 false negatives) were recorded between KK and the “gold standard,” while 37 discrepancies (0 false positives and 37 false negatives) were recorded for POC-CCA.

The overall sensitivity of KK (57.6%; 95% CI, 49.1–65.8) was significantly lower than that of POC-CCA (73.2%; 95% CI, 65.0–80.4; ${\chi }_{m}^{2}$= 5.88, *p* = 0.015). There was moderate agreement between KK and the combined results (*k* = 0.599, *p* < 0.001, concordance 80.9%), and a substantial agreement between POC-CCA and the combined results (*k* = 0.753, *p* < 0.001, concordance 88.1%). It is important to note that when using Kato-Katz as the “gold standard,” the sensitivity and specificity of POC-CCA were (51.3%; 95% CI, 39.6–63.0) and (70.2%; 95% CI, 63.5–76.4), respectively.

We observed increased hesitancy by parents and caregivers to providing children’s stool samples, as compared to urine samples. This was due to cultural beliefs that associate stool with witchcraft, necessitating additional community engagement efforts.

PSAC are an important demographic group in schistosomiasis endemic areas. Due to their relatively short exposure to infection, they mostly carry light schistosome infections, as confirmed by our study. This requires the deployment of sensitive diagnostics to limit the risk of under-estimating disease prevalence. This is even more crucial as control programs are increasingly considering the inclusion of PSAC in treatment programs following the development of a child-friendly praziquantel formulation (N'Goran et al. 2023). Studies have shown that when the KK prevalence is less than 20%, POC-CCA prevalence is generally 3 to 6 times higher (Armoo et al. 2020; Kittur et al. 2016) and up to 8 times higher in low endemic areas (Assaré et al. 2018). We confirmed that POC-CCA is more sensitive than duplicate KK thick smears in comparison to a “gold standard” based on the combination of KK and POC-CCA positives. Our results align with studies from Côte d’Ivoire, Ghana, Kenya, Tanzania, and Uganda, which reported higher *S. mansoni* prevalence using POC-CCA among school children compared to KK (Adriko et al. 2014; Armoo et al. 2020; Coulibaly et al. 2013; Standley et al. 2010), and extend these findings to PSAC.

It is important to acknowledge that the specificity of the POC-CCA test can be compromised in infants (Casacuberta-Partal et al. 2021), and by cross-reactivity with other helminth infections, diuretic use (Ferreira et al. 2017), urinary tract infections, and hematuria (Rapid Medical Diagnostics 2019). These factors can lead to false-positive results, potentially overestimating the true prevalence of schistosomiasis (Ferreira et al. 2017).

We conclude that POC-CCA is a diagnostic tool well suited for resource-limited settings, such as remote rural areas, due to its user-friendly format and minimal infrastructure requirements. Urine sample collection for POC-CCA, particularly among PSAC, is less cumbersome and logistically simpler than stool collection. Furthermore, POC-CCA offers a potential advantage in detecting pre-patent *S. mansoni* infections, whereas the Kato-Katz method is limited to the detection of patent infections.

Collectively, these attributes render the POC-CCA a suitable and practical tool for the expeditious diagnosis of intestinal schistosomiasis in PSAC living in hard-to-reach low-resource settings, particularly in environments with high transmission risks that require community-level interventions to mitigate morbidity and transmission.

## Data Availability

No datasets were generated or analysed during the current study.
